# Targeting S100A4 with niclosamide attenuates inflammatory and profibrotic pathways in models of amyotrophic lateral sclerosis

**DOI:** 10.1186/s12974-021-02184-1

**Published:** 2021-06-12

**Authors:** Martina Milani, Eleonora Mammarella, Simona Rossi, Chiara Miele, Serena Lattante, Mario Sabatelli, Mauro Cozzolino, Nadia D’Ambrosi, Savina Apolloni

**Affiliations:** 1 Department of Biology, University of Rome “Tor Vergata”, Via della Ricerca Scientifica, 1, 00133 Rome, Italy; 2 Institute of Translational Pharmacology, CNR, 00133 Rome, Italy; 3 Unità Operativa Complessa di Genetica Medica, Fondazione Policlinico Universitario A. Gemelli IRCCS, 00168 Rome, Italy; 4 Sezione di Medicina Genomica, Università Cattolica del Sacro Cuore, 00168 Rome, Italy; 5 Unità Operativa Complessa di Neurologia, Fondazione Policlinico Universitario A. Gemelli IRCCS, 00168 Rome, Italy; 6 Centro Clinico NEMO, Fondazione Policlinico Universitario A. Gemelli IRCCS, 00168 Rome, Italy; 7 Sezione di Neurologia, Università Cattolica del Sacro Cuore, 00168 Rome, Italy

**Keywords:** ALS, S100A4, Fibroblasts, FUS, α-SMA, Neurodegeneration, Inflammation

## Abstract

**Background:**

An increasing number of studies evidences that amyotrophic lateral sclerosis (ALS) is characterized by extensive alterations in different cell types and in different regions besides the CNS. We previously reported the upregulation in ALS models of a gene called fibroblast-specific protein-1 or S100A4, recognized as a pro-inflammatory and profibrotic factor. Since inflammation and fibrosis are often mutual-sustaining events that contribute to establish a hostile environment for organ functions, the comprehension of the elements responsible for these interconnected pathways is crucial to disclose novel aspects involved in ALS pathology.

**Methods:**

Here, we employed fibroblasts derived from ALS patients harboring the *C9orf72* hexanucleotide repeat expansion and ALS patients with no mutations in known ALS-associated genes and we downregulated S100A4 using siRNA or the S100A4 transcriptional inhibitor niclosamide. Mice overexpressing human *FUS* were adopted to assess the effects of niclosamide in vivo on ALS pathology.

**Results:**

We demonstrated that S100A4 underlies impaired autophagy and a profibrotic phenotype, which characterize ALS fibroblasts. Indeed, its inhibition reduces inflammatory, autophagic, and profibrotic pathways in ALS fibroblasts, and interferes with different markers known as pathogenic in the disease, such as mTOR, SQSTM1/p62, STAT3, α-SMA, and NF-κB. Importantly, niclosamide in vivo treatment of ALS-FUS mice reduces the expression of S100A4, α-SMA, and PDGFRβ in the spinal cord, as well as gliosis in central and peripheral nervous tissues, together with axonal impairment and displays beneficial effects on muscle atrophy, by promoting muscle regeneration and reducing fibrosis.

**Conclusion:**

Our findings show that S100A4 has a role in ALS-related mechanisms, and that drugs such as niclosamide which are able to target inflammatory and fibrotic pathways could represent promising pharmacological tools for ALS.

**Supplementary Information:**

The online version contains supplementary material available at 10.1186/s12974-021-02184-1.

## Background

Amyotrophic lateral sclerosis (ALS) is a late-onset neurodegenerative disease characterized by progressive loss of motor neurons in the brain and spinal cord. It is the most common form of motor neuron disease [6], with an onset occurring at approximately 60 years old and patients surviving on average 3 years from diagnosis. Most cases of ALS are sporadic (sALS), while 60% of familial ALS can be attributed to pathogenic variants in four genes: *SOD1*, *TARDBP*, *FUS*, and *C9orf72* [37].

An increasing number of studies supports the concept that ALS is not a disease restricted to motor neuron pathology, but a disorder characterized by extensive involvement of the CNS, with documented causal roles exerted also by non-neuronal cells [2]. Moreover, alterations in non-nervous tissues, including skeletal muscles, adipose tissue, and even dermis have been extensively documented [42, 57, 73]. Fibroblasts from patients with ALS show indeed numerous abnormalities concerning autophagy, stress response [23, 40, 48, 52], and the stability of RNA transcripts related to oxidative phosphorylation, protein synthesis, and inflammation [65]. These peripheral cells therefore share common pathogenic pathways with different CNS resident cells and represent an accessible model for studying molecular, cellular, and genetic parameters of the pathology [51].

Literature data and our previous work reported an evident upregulation of a gene called fibroblast-specific protein-1 or S100A4, in different models of ALS disease. S100A4 mRNA was found strongly increased in the lumbar spinal cord from pre-symptomatic and end-stage SOD1-G93A rats [59], in astrocytes from pre-symptomatic SOD1-G37R mice [64] and is among the limited number of mRNAs displaying significant changes in their stability in both *C9orf72* and sALS fibroblasts [65]. Accordingly, we found that S100A4 protein is overexpressed mainly by astrocytes and microglia from SOD1-G93A rats and by fibroblasts from ALS patients carrying SOD1 mutations [59]. The functions of S100A4 can be diverse and tissue-dependent but it is recognized as a pro-inflammatory and profibrotic gene, even though in the CNS its role seems more controversial, as in acute models of neurodegeneration it has been associated with trophic effects [12]. In contrast with this beneficial role, we previously demonstrated that in activated primary microglia cells, the decrease of S100A4 obtained using its transcriptional inhibitor niclosamide is associated with a strong reduction of pro-inflammatory pathways [59]. Under this aspect, S100A4 promotes the release of cytokines at inflammatory sites and the remodeling of extracellular matrix components (ECM), and is a recognized inhibitor of autophagy, sustaining by this way inflammation and concomitant fibrotic events. Due to its properties, the protein has been implicated in the fibrosis of many organs, such as kidney, liver, lung, and heart [26]. In neurodegenerative conditions, including ALS, an interplay between fibrosis and inflammation in different organs and tissues is an emerging concept that relies on data showing alterations of the ECM components and remodeling enzymes, increase in fibrotic markers as TGF-β, as well as in profibrotic genes [11, 16, 33]. Hence, the comprehension of the elements responsible for the inflammatory and fibrotic pathways appears to be crucial to dissect novel aspects contributing to the pathology of ALS.

Niclosamide is an FDA-approved anti-helminthic drug, with considerable safety [18, 61, 68]. In the recent years, niclosamide has been repurposed for different diseases and preclinical validation proved that it has promising efficacy against solid cancers, rheumatoid arthritis, and fibrotic conditions, due to the potent anti-inflammatory and anti-fibrotic properties [7, 27, 61]. Niclosamide effects reside on its ability to target several signaling pathways, including S100A4, mammalian target of rapamycin (mTOR), signal transducer and activator of transcription 3 (STAT3), and nuclear factor-κB (NF-κB) [21, 49, 58, 70], which, interestingly, have been found to be dysregulated in ALS [32, 59, 69], suggesting its potential use to interfere with these altered mechanisms in the pathology.

In this study, we have analyzed the role of S100A4 in cellular pathways linked to human ALS-fibroblasts activation, such as mTOR, sequestosome 1 (SQSTM1/p62), NF-κB, α-smooth muscle actin (α-SMA), and N-cadherin. Moreover, we have tested niclosamide in vitro in ALS fibroblasts and in vivo in a transgenic mouse model of ALS overexpressing human FUS (hFUS), recapitulating pathological features of the disease, to understand its potential efficacy in ameliorating ALS pathology.

## Methods

### Patients

The study was approved by the ethics committee of the Università Cattolica del Sacro Cuore (Rome, Italy) on 30 July 2012, Prot nr. P740/CE/2012. A written informed consent was signed by all of the subjects. The diagnosis of ALS was made according to revise El Escorial/Airlie House Criteria. The presence of familiarity was deeply investigated. Patients with one or more affected relatives were diagnosed as familial ALS (fALS), while patients with no family history were classified as sporadic (sALS). Genetic analysis was performed on patients using massive parallel sequencing of genes associated to ALS, as previously described [24], and Repeat-Primed PCR was used to screen all patients for the *C9orf72* expansion [50]. Three patients harboring the *C9orf72* hexanucleotide repeat expansion (2 fALS and 1 sALS), one patient harboring the p.R51C *FUS* pathogenic variant (fALS), two patients carrying the p.Q303H and the p.A382T variants in *TARDBP* (both sALS) were included in the study as well as three ALS patients with no pathogenic variants in known ALS-associated genes (sALS) and five healthy controls.

### Fibroblast primary cultures

All experiments were carried out in accordance to the approved guidelines of the ethics committee of the Catholic University. A written informed consent was obtained from patients and from healthy donors. Skin biopsies were performed using a 4-mm punch on the distal leg of the patients at NEMO Clinical Centre (Rome, Italy). Primary human dermal fibroblasts were isolated, as previously described [59]. Skin samples were dissected, transferred to a cell culture flask, and cultured in BIO-AMF-2 complete medium (Biological Industries) in a 37 °C incubator. After the fibroblasts reached confluence, they were expanded up to 4th passage. Fibroblasts were maintained in Dulbecco’s modified Eagle’s medium (DMEM) supplemented with 20% fetal bovine serum (FBS, Euroclone) and 1% penicillin/streptomycin (Sigma-Aldrich) at 37 °C, 5% CO_2_.

### FUS transgenic mice

Adult Tg (Prnp-FUS) WT3Cshw/J mice expressing hemagglutinin-tagged human wild-type FUS (hFUS) were obtained from Jackson Laboratories. Animals were housed in our indoor animal facility at constant temperature (22 ± 1 °C) and relative humidity (50%) with 12-h light cycle (light 7 am–7 pm). Mice were maintained in hemizygosity on the same C57BL/6 genetic background. Hemizygous FUS mice were backcrossed to obtain homozygous mice, used as experimental subjects. Food and water were freely available. When animals showed symptoms of paralysis, wet food was given daily into the cages for easy access to nutrition and hydration. Mice were genotyped by PCR analysis of tissue extracts from tail tips. Hemizygous FUS mice were identified using PCR primers: Fwr5′-AGGGCTATTCCCAGCAGAG-3′, Rev5′-TGCTGCTGTTGTACTGGTTCT-3′. Homozygous FUS mice were genotyped by qPCR using the following primers: Fwr5′-GCCAGAACACAGGCTATGGAA-3′ and Rev5′-GTAAGACGATTGGGAGCTCTG-5′. All animal experiments complied with the ARRIVE guidelines and were carried out in accordance with the European Guidelines for the use of animals in research (2010/63/EU) and the requirements of Italian laws (D.L. 26/2014). The ethical procedure was approved by the Italian Ministry of Health. All efforts were made to minimize animal suffering and the number of animals necessary to produce reliable results.

### S100A4 silencing

Primary fibroblasts deriving from ALS patients with no pathogenic variants in known ALS-associated genes (*n* = 3, uvALS) and C9orf72 patients (*n* = 3, C9orf72) were seeded in 12-well plate at a density of 50,000 cells per well approximately 24 h before transfection and at the confluence of about 50%, the cells were transfected with two types of siRNAs for S100A4 (50 nM) (Thermo Fischer). A scrambled siRNA (100 nM) (Thermo Fischer) was used as a negative control. Transfection was performed using Metafectene (Biontex, Germany) following the manufacturer’s instructions. After transfection for 48 or 72 h, cells were harvested for further experiments. The experiments were repeated in triplicate.

### Niclosamide in vitro and in vivo treatment

The inhibitor of S100A4 niclosamide (2′,5-dichloro-4′-nitrosalicylanilide, Sigma-Aldrich) was solubilized in dimethyl sulfoxide (DMSO) for in vitro experiments. Control cells were treated with the equal amount of DMSO. Niclosamide was administered to fibroblasts deriving from ALS patients with no pathogenic variants in known ALS-associated genes (*n* = 3, uvALS) and C9orf72 patients (*n* = 3, C9orf72) at the dose of 1–5–10 μM for 72 h. At these concentrations, niclosamide did not interfere with fibroblasts cell viability. The experiments were performed in triplicate.

For the in vivo experiments, niclosamide (20 mg/kg/day, dissolved in Cremophor **®,** Sigma-Aldrich) was administered daily from post-natal day 25 via intraperitoneal (i.p.) injections to hFUS mice (*n* = 6), when hFUS mice showed first signs of destabilized gait [34]. Control vehicle mice (*n* = 6) were treated with the appropriate volume of solvent solution. Survival was determined by the loss of righting reflex within 20 s after laying the mouse on its side [1].

### Western blot

Ctrl (*n* = 5), C9orf72 (*n* = 3), FUS (*n* = 1), TARDBP (*n* = 2), and uvALS (*n* = 3) fibroblasts were lysed on plates in 2xLaemmli buffer and the lysates were boiled at 100 °C for 5 min. Spinal cords, sciatic nerves, and gastrocnemius muscles of *n* = 4 animals per group were dissected [1] and lysed in homogenization buffer (50 mM Tris HCl pH 7.4, 250 mM NaCl, 1 mM EDTA, 5 mM MgCl_2_, 1% Triton X-100, 0.25% Na-deoxycholate, 0.1% SDS, protease inhibitor cocktail from Sigma-Aldrich). After 2 × 10″ sonication cycles, samples were incubated on ice and then centrifuged at 15,000×*g* for 20′ at 4 °C. Supernatants were then quantified with Bradford protein assay (Bio-Rad) and resuspended in Laemmli Buffer before SDS-PAGE (Sigma-Aldrich). Proteins were separated on 10% SDS-PAGE and transferred to nitrocellulose membranes, followed by incubation with 5% skimmed milk for 1 h and with primary antibodies at 4 °C overnight. HRP-conjugated secondary antibodies (1:2,500, Jackson ImmunoResearch) were applied at RT for 1 h. ECL solution (Roche) was used for chemiluminescent detection. GAPDH was used as a control for equal loading. Following densitometry-based quantification and analysis using ImageJ software, the relative density of each identified protein was calculated.

### Immunofluorescence and confocal analysis

FUS mice and age-matched controls were euthanized by CO_2_ and decapitated. Spinal cords were immediately dissected and post-fixed in 4% paraformaldehyde (PFA) for 12 h, incubated in 30% sucrose in PBS solution for 24 h at 4 °C, and then cut into 30-μm-thick slices with a freezing cryostat. Lumbar spinal cord slices from *n* = 4 animals per group were blocked for 1 h in 10% normal donkey serum (NDS) in PBS, 0.3% Triton X-100, and then incubated 3 days at 4 °C with primary antibodies diluted in 2% NDS in PBS, 0.3% Triton X-100, and then for 3 h at room temperature with appropriate secondary antibody, diluted in the same solution. After two rinses, 10 min each in PBS, nuclei were stained with 1 μg/ml DAPI (Sigma-Aldrich) for 10 min.

Whole mount sciatic nerves from *n* = 4 animals per group were post-fixed in 4% PFA for 24 h, incubated with PBS at 4 °C for 48 h, and blocked with blocking buffer of 10% NDS in PBS, 0.3% Triton X-100 for 6 h at RT. Nerves were then incubated 3 days at 4 °C with primary antibodies diluted in 2% NDS in PBS, 0.3% Triton X-100, and then for 3 h at RT with appropriate secondary antibody, diluted in the same solution. After two rinses, 10 min each in PBS, nuclei were stained with 1 μg/ml DAPI for 10 min. Images were visualized by Nikon Eclipse TE200 epifluorescence microscope (Nikon, Florence, Italy) connected to a CCD camera. Images were captured under constant exposure time, gain, and offset. After creating a region of interest, background was subtracted, and the average pixel intensity was determined. All image quantifications were done using ImageJ software (NIH, Bethesda, USA).

### Antibodies

Immunofluorescences (IF) and immunoblots (WB) were performed with the following primary antibodies: anti-rabbit S100A4 (1:500-IF, 1:1000-WB, Millipore), anti-rabbit mTOR and phospho-mTOR (1:100-WB, Cell Signaling), anti-mouse SQSTM1/p62 (1:1000-WB, Abcam), anti-rabbit NF-kB and phospho-NF-kB (1:1000-WB, Cell Signaling), anti-rabbit STAT3 (1:1000-WB, Cell Signaling), anti-rabbit phospho-STAT3 (1:2000-WB, Cell Signaling), anti-rabbit α-SMA (1:1000-WB, 1:500-IF, GeneTex), anti-rabbit N-cadherin (1:1000-WB, GeneTex), anti-mouse glial fibrillary acidic protein (GFAP) (1:500-IF, 1:1000-WB, Cell Signaling), anti-rabbit β-III Tubulin (1:500-IF, Cell Signaling), anti-mouse MyoG (1:200-WB, Hybridoma Bank, USA), anti-mouse platelet-derived growth factor receptor β (PDGFR-β) (1:250-WB, 1:500-IF, Santa Cruz), and anti-mouse GAPDH (1:10000-WB, Calbiochem). Secondary immunoblot antibodies for WB were anti-rabbit (1:2500) and anti-mouse (1:5000) IgG peroxidase-conjugated from Bio-Rad Laboratories (Hercules, CA, USA). Secondary fluorescent antibodies for IF were Alexa-Flour 488-Donkey anti-rabbit (1:200) and Cy3-Donkey anti-mouse (1:200) from Jackson ImmunoResearch Laboratories (West Grove, PA, USA). DAPI was used to stain nuclei (1:1000, Thermo Fisher Scientific, Waltham, MA, USA).

### RT-PCR and qPCR

Total RNA from C9orf72 (*n* = 3) and uvALS (*n* = 3) fibroblasts and from gastrocnemius skeletal muscle tissue (*n* = 4 animals per group) was extracted with Trizol (Invitrogen) using a standard protocol, and treated with RNAse-free DNase (Promega), according to the manufacturer’s instruction. RNA was retro-transcribed using random primers with Im-Prom II reverse transcription system (Promega), following the manufacturer’s indication. qPCR was performed with iTaq Universal SYBR Green Supermix (Bio-Rad) using 20 ng cDNA and 350 nM of specific primers, following manufacturer’s indications. qPCR reactions were performed using the CFX Connect Real-Time PCR Detection System (Bio-Rad), and Cq values were determined from the system software using ‘single threshold’ mode. Relative expression values were normalized to the housekeeping genes GAPDH or β-actin for human fibroblasts and murine muscle tissues, respectively. The primers used for human fibroblasts were GAPDH FOR: TCTTTTGCGTCGCCAGCCGAG, GAPDH REV: TGACCAGGCGCCCAATACGAC; S100A4 FOR: GTACTCGGGCAAAGAGGGTG, S100A4 REV: GCTTCATCTGTCCTTTTCCCC; α-SMA FOR: ACTGCCTTGGTGTGTGACAA, α-SMA REV: CACCATCACCCCCTGATGTC. The primers used for murine muscle tissues were β-actin FOR: CTAAGGCCAACCGTGAAAAG, β-actin REV: ACCAGAGGCATACAGGGACA; MyoG FOR: GTCCCAACCCAGGAGATCAT, MyoG REV: CCACGATGGACGTAAGGGAG; MyoD FOR: AGCACTACAGTGGCGACTCA, MyoD REV: GCTCCACTATGCTGGACAGG; Pax7 FOR: TCAAGCCAGGAGACAGCTTG, Pax7 REV: AGGTAATCAACAGCAGTTTGGC; MHC FOR: ACATATCAGAGTGAGGAG, MHC REV: TCTTGTAGGACTTGACTT.

### Statistics

Data are reported as mean ± standard error of the mean (SEM). Statistical differences were verified by two-tailed student’s *t* test if the normality test was passed, or by the Mann–Whitney rank sum test, if the normality test failed. One-way analysis of variance (ANOVA) followed by post hoc Tukey’s was used for multiple comparisons. The software package GraphPad Prism 6.0 (GraphPad Software, San Diego, CA, USA) was used for all statistical analysis with differences considered significant for *p* < 0.05. Animals were randomly used for experiments. The sample sizes were chosen on the basis of similar experiments reported in our previous papers and papers published by other groups [1, 28, 48, 55, 59].

## Results

### ALS fibroblasts show aberrant levels of S100A4, mTOR, SQSTM1/p62, and NF-κB

**Fig. 1:**
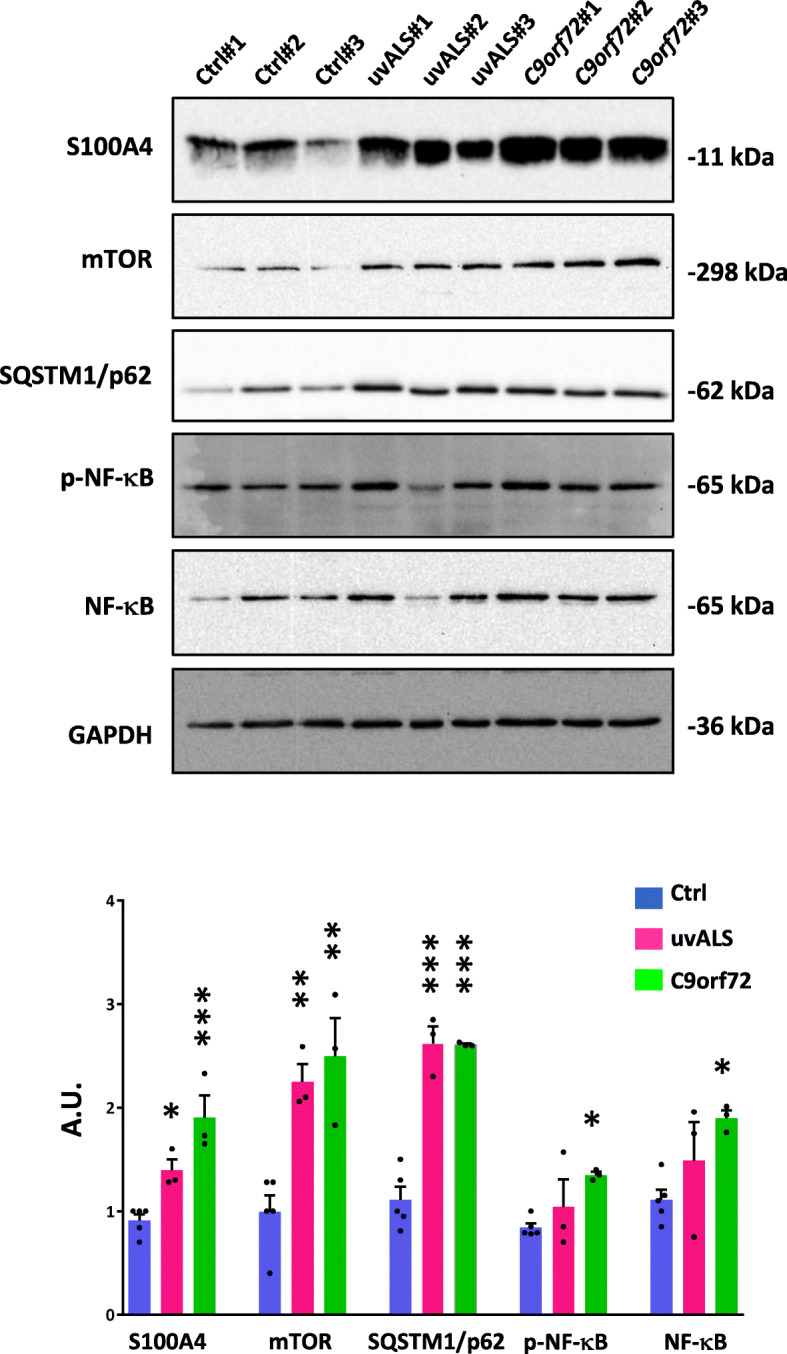
ALS-derived fibroblasts show increased activation-related pathways. Protein lysates of fibroblasts from controls (Ctrl), ALS patients with no known ALS variants (uvALS), and C9orf72 ALS patients were analyzed by Western blot using anti-S100A4, anti-mTOR, anti-SQSTM1/p62, anti-p-NF-κB and anti-NF-κB. GAPDH was used to normalize samples. The expression levels were calculated by densitometric analyses. Data represent mean ± SEM. *n* = 5 individuals per control fibroblasts and *n* = 3 per each group of ALS fibroblasts, experiments repeated in triplicate; One-way ANOVA with Tukey correction between Ctrl and uvALS and C9orf72. *F* value (DFn, DFd): (2, 8) = 18.22 (S100A4), (2, 8) = 14.19 (mTOR), (2, 8) = 51.36 (SQSTM1/p62), (2, 8) = 4.103 (p-NFκB), (2, 8) = 4.445 (NFκB); *p < 0.05,**p < 0.01, and ***p < 0.001 vs. control fibroblasts

In a previous study, we demonstrated that S100A4 was increased in fibroblasts from patients with different SOD1 pathogenic variants [59]. To investigate whether an augmented expression of S100A4 is a common trait of fibroblasts derived from patients with ALS, we have analyzed the protein expression in primary fibroblasts from ALS patients without known variants in ALS-associated genes, and from patients carrying pathogenic *C9orf72* expansions, the most common cause of familial and sporadic ALS found to date. As shown, primary fibroblasts derived from both groups of patients display a strong increase in S100A4 protein levels, compared with those obtained from healthy subjects (Fig. 1). Furthermore, S100A4 shows a trend to increase also in a fibroblast line derived from a patient carrying the *FUS* p.R521C pathogenic variant (Additional file 1: Figure S1a) and from patients with the *TARDBP* p.Q303H and p.A382T mutations (Additional file 1: Figure S1b), suggesting that S100A4 is upregulated in fibroblasts regardless of the ALS condition and gene mutation carried.

Since the overexpression of S100A4 is correlated with autophagy impairment and inflammation, we also analyzed key markers related to these pathways in ALS fibroblasts. Cells from ALS patients show increased mTOR expression and an accumulation of SQSTM1/p62, compared to cells from healthy controls (Fig. 1). Moreover, although fibroblasts from ALS patients with no known ALS variants (uvALS) do not show significant differences in both total and p-NF-κB levels compared to controls, fibroblasts carrying the *C9orf72* expansions display increased total and activated NF-κB (Fig. 1). These findings indicate that ALS-derived primary fibroblasts show features of autophagic and inflammatory pathway alterations, which may suggest an activated phenotype.

### S100A4 silencing inhibits activation markers in ALS fibroblasts

**Fig. 2:**
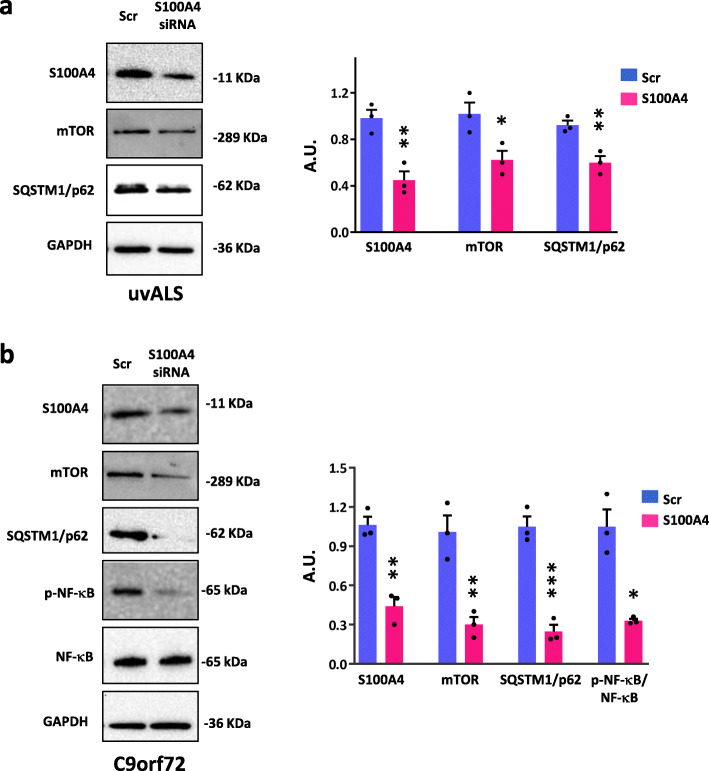
S100A4 silencing inhibits activation markers of ALS fibroblasts. Fibroblasts from ALS patients with no known ALS variants (uvALS) and C9orf72 ALS patients were treated with S100A4 siRNA and scrambled siRNA (Scr) and then harvested after 72 h of transfection. **a** Protein lysates of uvALS fibroblasts were subjected to western blot using anti-S100A4, anti-mTOR, anti-SQSTM1/p62. GAPDH was used to normalize samples. Values are mean ± SEM, *n* = 3 individuals, experiments repeated in triplicate; two-tailed *t* test. *t* value: 5.037, degrees of freedom: 4 (S100A4); *t* value: 3.141, degrees of freedom: 4 (mTOR); *t* value: 4.630, degrees of freedom: 4 (SQSTM1/p62). *p < 0.05, **p < 0.01, and ***p < 0.001 vs. scrambled siRNA. **b** Protein lysates of C9orf72 fibroblasts were subjected to western blot using anti-S100A4, anti-mTOR, anti-SQSTM1/p62, anti-NF-κB, and anti-p-NF-κB. GAPDH was used to normalize samples. Values are mean ± SEM, *n* = 3 individuals, experiments repeated in triplicate; two-tailed *t* test. *t* value: 6.476, degrees of freedom: 4 (S100A4); *t* value: 5.183, degrees of freedom: 4 (mTOR); *t* value: 8.708, degrees of freedom: 4 (SQSTM1/p62); two-tailed *t* test with Welch’s correction. *t* value: 5.407, degrees of freedom: 2.053 (p-NFκB/NFκb). *p < 0.05, **p < 0.01, and ***p < 0.001 vs. scrambled siRNA

In order to directly assess the contribution of S100A4 in supporting the autophagic and inflammatory dysregulated pathways shown by ALS fibroblasts, we silenced S100A4 expression in patient-derived cells. We found that a 60% downregulation of S100A4 is sufficient to strongly decrease the levels of mTOR and SQSTM1/p62 proteins in fibroblasts from ALS patients (Fig. 2a, b), as well as the expression of p-NF-κB in C9orf72 cells (Fig. 2b), with respect to scramble silenced cells.

**Fig. 3:**
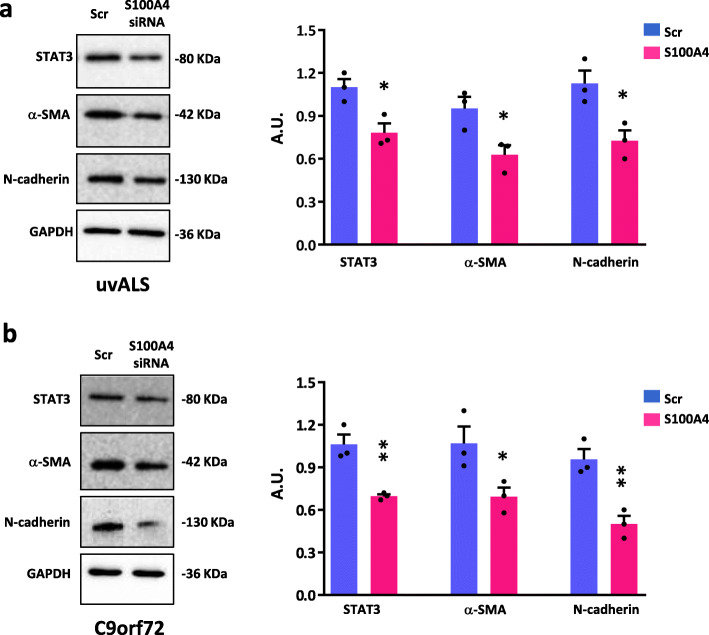
S100A4 is involved in profibrotic pathways. Fibroblasts from ALS patients with no known ALS variants (uvALS) and C9orf72 ALS patients were transfected with S100A4 siRNA and scrambled siRNA (Scr). Cells were lysed after 72 h of transfection. **a** Protein lysates of uvALS fibroblasts were assayed by Western blot with anti-STAT3, anti-α-SMA, anti-N-cadherin. The levels of GAPDH expression were used as loading control. Values are mean ± SEM, *n* = 3 individuals, experiments repeated in triplicate; two-tailed *t* test. *t* value: 3.687, degrees of freedom: 4 (STAT3); *t* value: 3.169, degrees of freedom: 4 (α-SMA); *t* value: 3.474, degrees of freedom: 4 (N-cadherin). **b** Protein lysates of C9orf72 ALS fibroblasts were assayed by Western blot with anti-STAT3, anti-α-SMA, anti-N-cadherin. The levels of GAPDH expression were used as loading control. Values are mean ± SEM, *n* = 3 individuals, experiments repeated in triplicate; two-tailed *t* test. *t* value: 5.066, degrees of freedom: 4 (STAT3); *t* value: 2.812, degrees of freedom: 4 (α-SMA); *t* value: 4.940, degrees of freedom: 4 (N-cadherin). *p < 0.05 and **p < 0.01 vs. scrambled siRNA

The transformation of fibroblasts into activated cells as profibrotic myofibroblasts is characterized by the upregulation of several distinctive markers, including S100A4, α-SMA, N-cadherin, and by the activation of the STAT3 pathway. To explore whether the inhibition of S100A4 may affect the expression of these markers, we adopted the conditions of S100A4 silencing described before, and tested the levels of these proteins. As shown, S100A4 silencing leads to a decreased expression of STAT3, N-cadherin, and α-SMA, in ALS fibroblasts (Fig. 3a, b) compared to scramble silenced cells. These findings thus suggest that S100A4 is directly involved in aberrant pathways related to autophagy and inflammation and contributes to the phenotypic transition of ALS fibroblasts toward a profibrotic and activated state.

### Niclosamide decreases S100A4, mTOR, and profibrotic markers in ALS fibroblasts

**Fig. 4:**
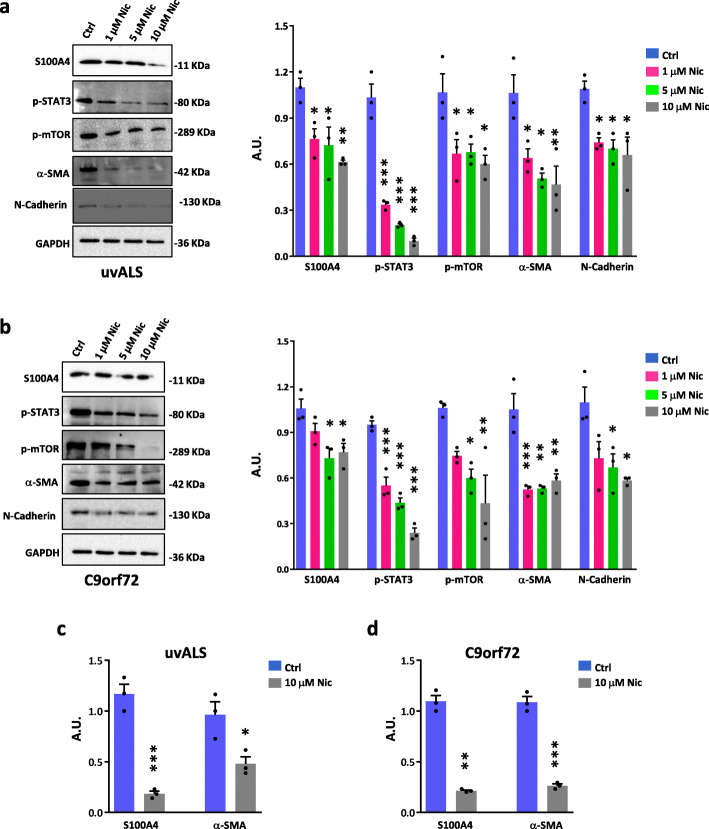
Niclosamide induces phenotypic changes in ALS fibroblasts. Fibroblasts from ALS patients with no known ALS variants (uvALS) and C9orf72 ALS patients were treated for 72 h with three different concentration of niclosamide (Nic): 1 μM, 5 μM, and 10 μM. **a** Protein expression levels of uvALS fibroblasts were analyzed by western blot with anti-S100A4, anti-p-STAT3, anti-p-mTOR, anti-α-SMA, and anti-N-cadherin. GAPDH was used as a loading control. Data represent mean ± SEM. *n* = 3 individuals, experiments repeated in triplicate. One-way ANOVA with Tukey correction between multiple comparisons. *F* value (DFn, DFd): (3,8) = 8.053 (S100A4), (3,8) = 83.52 (p-STAT3), (3,8) = 6.182 (p-MTOR), (3,8) = 9.100 (α-SMA), (3,8) = 7.665 (N-cadherin). *p < 0.05, **p < 0.01, and ***p < 0.001 vs. untreated (Ctrl) cells. **b** Protein expression levels of C9orf72 fibroblasts were analyzed by Western blot with anti-S100A4, anti-p-STAT3, anti-p-mTOR, anti-α-SMA, and anti-N-cadherin. GAPDH was used as a loading control. Data represent mean ± SEM. *n* = 3 individuals, experiments repeated in duplicate. One-way ANOVA with Tukey correction between multiple comparisons. *F* value (DFn, DFd): (3,8) = 6.264 (S100A4), (3,8) = 63.13 (p-STAT3), (3,8) = 7.163 (p-MTOR), (3,8) = 19.08 (α-SMA), (3,8) = 6.757 (N-cadherin). *p < 0.05, **p < 0.01, and ***p < 0.001 vs. untreated (Ctrl) cells. **c** mRNA levels of S100A4 and α-SMA in uvALS fibroblasts treated with 10 μM niclosamide for 72 h. Data are normalized to GAPDH. Values are mean ± SEM, *n* = 3 individuals, experiments repeated in triplicate; two-tailed *t* test. *t* value: 9.961, degrees of freedom: 4 (S100A4); *t* value: 3.331, degrees of freedom: 4 (α-SMA). *p < 0.05 and **p < 0.001 vs. untreated (Ctrl) cells. **d** mRNA levels of S100A4 and α-SMA in C9orf72 fibroblasts treated with 10 μM niclosamide for 72 h. Data are normalized to GAPDH. Values are mean ± SEM, *n* = 3 individuals, experiments repeated in triplicate; two-tailed *t* test with Welch’s correction. *t* value: 14.52, degrees of freedom: 2.094 (S100A4); two-tailed *t* test. *t* value: 14.16, degrees of freedom: 4 (α-SMA). *p < 0.01 and **p < 0.001 vs. untreated (Ctrl) cells

Previous studies reported that niclosamide, a pleiotropic drug recognized as a transcriptional inhibitor of S100A4, can induce canonical autophagy via feedback downregulation of mTOR [30] and can exert a potent inhibitory activity on STAT3 [9], in line with its well-recognized anti-fibrotic action [7]. Thus, we tested the effects of different doses of niclosamide on ALS fibroblasts to evaluate its ability to reverse the aberrant pathways observed. Niclosamide treatment decreases S100A4, α-SMA, N-cadherin, p-mTOR, and p-STAT3 levels in fibroblasts from ALS patients (Fig. 4a, b). Moreover, in both patient-derived cells, niclosamide decreases mRNA levels of S100A4 and α-SMA (Fig. 4c, d), without significantly affecting N-cadherin transcript (data not shown). Overall, these data show that niclosamide reverses several parameters linked to inflammation, impaired autophagy, fibrosis and activation of ALS fibroblasts.

### Niclosamide reduces ALS pathology in transgenic mice carrying hFUS mutation

**Fig. 5:**
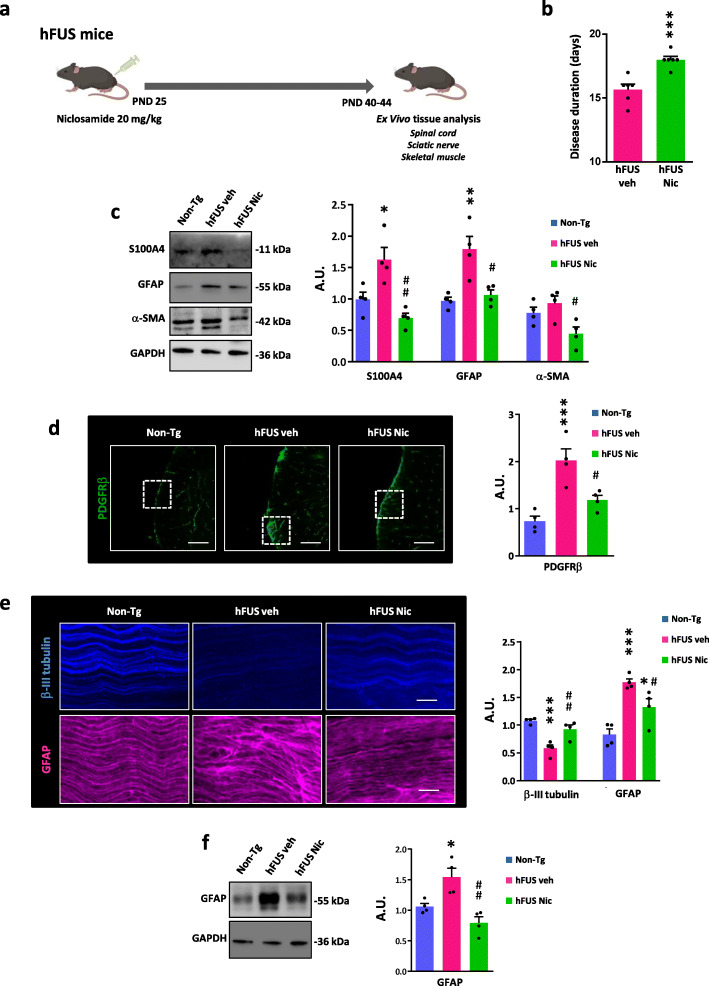
Niclosamide ameliorates pathology in hFUS symptomatic mice. **a** Schematic illustration of niclosamide treatment in hFUS mice. Male mice were intraperitoneally injected daily with 20 mg/kg niclosamide from postnatal day (PND) 25 until death and spinal cord, sciatic nerves and skeletal muscles tissues were then analysed. **b** Niclosamide-treated hFUS mice (hFUS Nic) show a significant difference in the disease duration with respect to vehicle-treated hFUS mice (hFUS veh). Data are presented as means ± SEM. *n* = 6 mice/group. Two-tailed *t* test. *t* value: 4.719, degrees of freedom: 10. ***p < 0.001 vs. vehicle-treated hFUS mice. **c** Protein lysates from lumbar spinal cord of non-transgenic (Non-Tg) (~ 40 days), vehicle (hFUS veh) and niclosamide-treated hFUS mice (hFUS nic) at end stage of the disease were assayed by western blot with anti-GFAP, anti-S100A4, and anti-α-SMA. Data represent mean ± SEM of *n* = 4 mice/group. One-way ANOVA with Tukey correction between Non-Tg, hFUS veh and hFUS nic. *F* value (DFn, DFd): (2, 9) = 11.26 (GFAP), (2, 9) = 11.73 (S100A4), (2, 9) = 5.721 (a-SMA). *p < 0.05 and **p < 0.01 vs. Non-Tg mice or ^#^*p* < 0.05 and ^##^*p* < 0.01 vs. hFUS veh mice. **d** Representative fluorescence images of PDGFRβ (green) in the lumbar spinal cord of Non-Tg, hFUS veh, and hFUS nic mice at end stage of the disease. Scale bars: 50 μm. Immunofluorescence intensities were calculated by densitometric analyses. Data represent mean ± SEM. *n* = 4 mice/group, four sections per animal. One-way ANOVA with Tukey correction between Non-Tg, hFUS veh, and hFUS nic. *F* value (DFn, DFd): (2, 9) = 15.75 ***p < 0.001 vs. Non-Tg mice or ^#^*p* < 0.05 vs. hFUS veh mice. **e** Representative fluorescence images of β-III tubulin (blue) and GFAP (purple) in the sciatic nerves of Non-Tg, hFUS veh, and hFUS nic mice at end stage of the disease. Scale bars: 50 μm. Immunofluorescence intensities were calculated by densitometric analyses. Data represent mean ± SEM. *n* = 4 mice/group, four sections per animal. One-way ANOVA with Tukey correction between Non-Tg, hFUS veh, and hFUS nic. *F* value (DFn, DFd): (2, 9) = 18.48 (β-III tubulin), (2, 9) = 17.94 (GFAP). *p < 0.05; **p < 0.01; and ***p < 0.001 vs. Non-Tg mice or ^#^*p* < 0.05 and ^##^*p* < 0.01 vs. hFUS veh mice. **f** Protein lysates from sciatic nerves of Non-Tg, hFUS veh, and hFUS Nic mice at end stage of the disease were assayed by western blot with anti-GFAP. GAPDH served as loading control. Relative densitometric values are reported on the right. Data represent mean ± SEM of *n* = 4 mice/group. One-way ANOVA with Tukey correction between Non-Tg, hFUS veh, and hFUS nic. *F* value (DFn, DFd): (2, 9) = 12.72. *p < 0.05 vs. Non-Tg mice or ^##^*p* < 0.01 vs. hFUS veh mice

It is established that S100A4 is upregulated in mutant SOD1 transgenic rat and mouse models of ALS during the disease course [59, 64]. To understand whether the increase of S100A4 is a common trait in rodent models originating from different ALS-associated genes, here we analyzed its protein expression in wild-type human FUS-overexpressing mice. The hFUS model recapitulates all key features of ALS such as motor neuron degeneration, muscle atrophy, physiological decline, cachexia, and neuroinflammation [28]. Notably, S100A4 is increased in the lumbar spinal cord (Additional file 1: Figure S2a and b) of end stage hFUS mice. This result indicates that the protein expression is commonly deregulated in different in vivo models of ALS, and prompted us to test the effects of S100A4 inhibition on disease phenotypes. To this aim, we treated hFUS mice with niclosamide at the dose of 20 mg/kg [56, 72], starting from the early symptom onset and analyzed the efficacy of the compound to restore several aberrant parameters occurring in these mice (Fig. 5a). At the employed dose, niclosamide slightly but significantly increases the disease duration, compared to vehicle-treated mice (Fig. 5b). Further, spinal cord pathology is improved, as indicated by the decrease in the levels of S100A4, as well as of GFAP and α-SMA in hFUS treated mice, compared to vehicle-treated mice (Fig. 5c). As shown in Fig. 5d, while spinal cord sections from non-Tg mice show PDGFRβ-positive cells (indicating cells of mesenchymal origin) in the meninges and around blood vessels, in hFUS mice PDGFRβ staining is infiltrated into the white matter parenchyma, suggesting the presence of fibrotic regions. Interestingly, PDGFRβ-positive infiltrates in the white matter are reduced after niclosamide treatment (Fig. 5d). Next, since peripheral nerves are strongly affected in the hFUS model [28], we investigated the effects of niclosamide on sciatic nerves. As observed, the sciatic nerve of hFUS mice shows an axonal impairment as demonstrated by the decrease in β-III tubulin-positive fibers and the concomitant upregulation of GFAP, in accordance with a Wallerian degeneration, evidencing a disorganization of Schwann cells compared with sciatic nerve from control littermate mice (non-Tg) (Fig. 5e, f). Niclosamide treatment partially restores the levels of β-III tubulin and GFAP and, in the niclosamide group both β-III tubulin and GFAP expression appear flatter and less frayed with respect to the vehicle group, suggesting that the treatment ameliorates axonal impairment in hFUS mice sciatic nerves (Fig. 5e, f).

### Niclosamide ameliorates muscle atrophy and fibrosis in hFUS mice

**Fig. 6:**
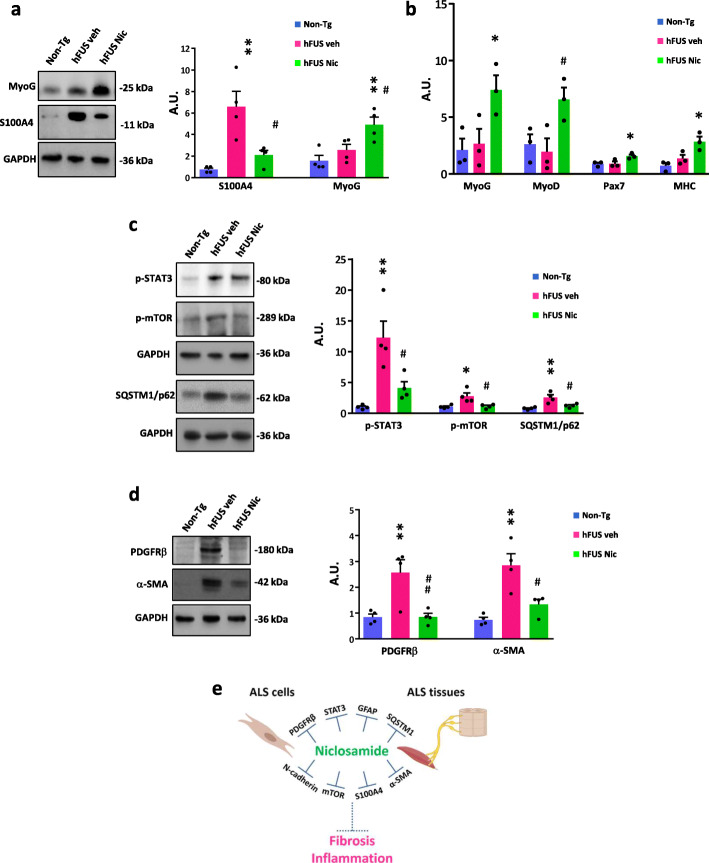
Niclosamide reduces profibrotic and inflammatory pathways in the skeletal muscles of hFUS symptomatic mice. Protein lysates from gastrocnemius muscles of non-transgenic (Non-Tg) ~ 40 days, vehicle-treated (hFUS veh) and niclosamide-treated hFUS mice (hFUS nic) at end stage of disease were subjected to Western blot with anti-S100A4 and anti-MyoG (**a**); with anti-p-STAT3, anti-p-mTOR, and anti-SQSTM1/p62 (**c**); and with anti-PDGFR-β and α-SMA (**d**). GAPDH served as loading control, *n* = 4 mice/group. Alongside the blots are reported the relative densitometric values. Data represent mean ± SEM of *n* = 4 mice/group. One-way ANOVA with Tukey correction between Non-Tg, hFUS veh and hFUS nic. *F* value (DFn, DFd): (2, 9)= 12.65 (S100A4), (2, 9) = 8.492 (MyoG), (2, 9) = 12.21 (p-STAT3), (2, 9) = 8.118 (p-mTOR), (2, 9) = 12.04 (SQSTM1/p62), (2, 9) = 10.07 (PDGFR-β), (2, 9) = 14.17 (α-SMA). *p < 0.05 and **p < 0.01 vs. Non-Tg mice or #*p* < 0.05 and ##*p* < 0.01 vs. hFUS veh mice. **b** Real time PCR for MyoG, MyoD, Pax7, and MHC in the gastrocnemius muscles of non-transgenic (Non-Tg) ~ 40 days, vehicle-treated (hFUS veh) and niclosamide-treated hFUS mice (hFUS nic) at end stage of disease. Data are normalized to β-actin and expressed as mean ± S.E.M. of *n* = 3 mice/group, experiments performed in triplicate. One-way ANOVA with Tukey correction between Non-Tg, hFUS veh, and hFUS nic. *F* value (DFn, DFd): (2, 6) = 5.790 (MyoG), (2, 6) = 5.648 (MyoD), (2, 6) = 6.155 (Pax7), (2, 6) = 9.657 (MHC). *p < 0.05 vs. Non-Tg mice or #*p* < 0.05 vs. hFUS veh mice. **e** Schematic illustration of niclosamide inhibition on multiple signalling pathways, i.e., S100A4, mTOR, SQSTM1/p62, NF-κB, STAT3, α-SMA, N-cadherin, and PDGFRβ dysregulated in ALS patients derived-cells and in ALS mice tissues with overall decrease of inflammatory and fibrotic state

Finally, we explored the effects of niclosamide treatment on hFUS skeletal muscle pathology. At first, we found that hFUS mice show a strong increase in S100A4 protein in the gastrocnemius muscle compared to healthy mice and that niclosamide strongly inhibits its level (Fig. 6a). We next assessed the expression of a key myogenic transcription factor MyoG a marker of muscle differentiation [14] and we found that, compared to vehicle-treated mice, niclosamide administration increases MyoG expression (Fig. 6a), suggesting an improved myogenic differentiation. To support the observations that niclosamide ameliorates muscle atrophy, we performed a qRT-PCR gene expression analysis of the myogenic factors MyoD, Pax7, and myosin heavy chain 3 (MHC) in addition to MyoG [14, 66], demonstrating a strong increase of all these markers in niclosamide treated hFUS mice (Fig. 6b). Importantly, muscles from hFUS mice show increased levels of p-STAT3, p-mTOR, and SQSTM1/p62 (Fig. 6c) which suggest that pathways involved in skeletal muscle atrophy and fibrosis [10, 32] are activated in this tissue; further, profibrotic markers, such as PDGFRβ and α-SMA, are also upregulated in hFUS compared to non-Tg mice (Fig. 6d). Remarkably, niclosamide decreases the expression of all aforementioned molecules (Fig. 6c, d), indicating that, besides modulating S100A4, it targets multiple signaling pathways (Fig. 6e).

## Discussion

In this work, we provide evidence for the contribution of S100A4 in ALS pathogenesis and the potential repurposing of niclosamide for preclinical trials in the disease. Indeed, we have demonstrated here that S100A4 is upregulated in fibroblasts derived from different ALS patients as well as in the ALS model represented by hFUS mice. These data are consistent with our previous results, showing an increase of S100A4 in the microglia and astrocytes from a SOD1 rat model in vivo and in mutant SOD1 fibroblasts in vitro [59] and suggest that an increased level of S100A4 is a common pathological trait of ALS, shared by different experimental models and disease-associated gene variants. Remarkably, in a recent paper, S100A4 mRNA was identified together with other 333 transcripts, out of 22,977 annotated transcripts, among those whose stability is altered in *C9orf72* ALS and sALS fibroblasts [65], sustaining our hypothesis that S100A4 dysregulation is a pathological hallmark of the disease shared by different cell types independently from their genetic variants and thus possibly reflecting a general reactive cellular state. Furthermore, recent studies show that S100A4 is one of the 88 upregulated genes of the pan-neurodegenerative signature obtained from the meta-analysis of human CNS transcriptomic datasets from Alzheimer’s and Lewy body diseases and ALS-frontotemporal dementia patients, suggesting that S100A4 represents a common substrate driving neurodegeneration [38, 39].

S100A4 belongs to the S100 superfamily, constituted by small proteins that are generally secreted by cells under stressful conditions, and that are undergoing extensive research as biomarkers in different fields, such as oncology, cardiology, fibrosis, and inflammation as well as brain injury pathologies [15, 63]. Within the limitations of our analysis, which is mainly based on a small number of patients for each ALS subgroup, our results, showing S100A4 upregulation as a common hallmark in ALS fibroblasts, make S100A4 a potential candidate to be tested as a biomarker in the disease. Recently, primary skin fibroblasts derived from patients have been extensively used as a model to study ALS because they share pathological alterations with neural cells, concerning stress–responses, autophagy, inflammation, and RNA processing [51]. Under this aspect, they are useful tools to explore new pathogenic mechanisms and perform preliminary assessments of novel potential treatments. Since they display overt limitations, in further experiments patient-derived models, including iPSC-derived neurons and glia, as well as transdifferentiated somatic cells [35, 60], should be necessary to examine the specific role of S100A4 in the different ALS cell phenotypes.

Moreover, fibroblasts represent a cell type that can become resident in the nervous system during inflammation [45], as well as in skeletal muscle. Indeed, activated fibroblasts (deriving from endothelial cells, pericytes, immune cells) can be accounted as cellular players in the development of fibrosis and inflammation during several neurodegenerative conditions, including ALS [5, 11, 43, 71]. Thus, the identification of the molecules and pathways involved in the transition of fibroblasts from a quiescent to an activated phenotype might unveil pathogenic mechanisms occurring in CNS and peripheral tissues. Fibroblast activation could represent a response to counteract and repair damage, that eventually evolves into a detrimental process as disease accelerates, leading to a non-permissive environment to cell regeneration. As recently reported [13], the robust fibrotic response to both injury and inflammation may be a common pathogenic mechanism across many different neurological disorders that should stimulate future research.

Extensive studies have shown that the transformation into activated fibroblasts is an extremely complex process involving numerous signaling pathways and that depends on the physiological or pathological status of the cells and on their specific cellular contexts [44]. Among these, recent studies indicate that mTOR and the substrate of autophagy SQSTM1/p62 contribute to mesenchymal transition and that autophagy enhancers can attenuate fibroblast activation [29, 44, 46]. Moreover, the NF-κB pathway also plays an important role in inducing a myofibroblast-like phenotype, especially under inflammatory conditions, elicited for instance by TNF-α or IL-6 [19]. We have demonstrated here that high levels of S100A4 in ALS-fibroblasts correlate with signs of impaired autophagy and inflammation, as suggested by high expression of mTOR, SQSTM1/p62, and NF-κB. It is well known that an increase in S100A4 characterizes profibrotic activated fibroblasts, as those induced by TGFβ [67]. Therefore, the dysregulation of these markers points to an activated pro-inflammatory and fibrotic phenotype of fibroblasts derived from patients with ALS compared to cells from healthy donors.

Our results show that the molecular changes characterizing the activated state of ALS-fibroblasts are limited when the expression of S100A4 is knocked-down, demonstrating that S100A4 is not only a marker of activation, but a necessary driver of the aberrant phenotypes of ALS-fibroblasts. Consistently, S100A4 is a well-known activator of the NF-κB axis [26] and its downregulation promotes autophagy, while its overexpression inhibits starvation-induced autophagic pathways [20, 54]. Remarkably, the depletion of S100A4 decreases the levels of the typical fibrotic markers α-SMA and N-cadherin and of the pro-fibrotic factor STAT3. STAT3 contributes to fibrosis by inducing the production of ECM, generally sustaining the differentiation of organ resident cells via canonical and non-canonical pathways. Indeed, several lines of evidence report the fundamental role of STAT3 in fibroblast plasticity in different tissues [8, 22], where the inhibition of its signaling pathway attenuates fibrosis by decreasing several markers, among which α-SMA [41] and N-cadherin [31]. Since S100A4 is a known inducer of JAK/STAT pathway, it is possible that S100A4 knock-down can indirectly decrease the levels of the fibrotic molecules α-SMA and N-cadherin through the inhibition of STAT3. Nevertheless, we may not exclude a direct effect of S100A4 depletion on these markers, in particular on α-SMA, through the c-Myb and sphingosine-1-phosphate (S1P pathway) [26]. Independently of the molecular pathways, we have shown here that the specific inhibition of S100A4 can revert a pathological phenotype of ALS-fibroblasts, suggesting a role for the protein in sustaining harmful mechanisms in ALS.

To evaluate the effects of S100A4 downregulation by a pharmacological approach, we employed niclosamide, a well-known S100A4 transcriptional inhibitor, which is also recognized as a multi-target drug that promotes autophagy and inhibits STAT3 and NF-κB and as a potent blocker of fibrotic signaling [4]. Our results demonstrate that the drug is able to reduce inflammatory/autophagic/fibrotic pathways in ALS fibroblasts, thereby interfering with different mechanisms characterized as pathogenic in ALS. Most interestingly, our in vivo results demonstrate that niclosamide relieves ALS-related pathological features in spinal cord, sciatic nerve, and skeletal muscle of hFUS mice. Central and peripheral nerve pathology with inflammation and fibrosis is a major harmful mechanism contributing to degeneration [25, 74]. In ALS, neuronal regeneration and axonal growth may be limited by a hostile environment characterized by extensive gliosis and aberrant remodeling of ECM components [11]. Accordingly, gene ontology analysis of differently expressed genes in the spinal cord of hFUS mice show ECM matrix disorganization and increased expression of proteoglycans [47, 53]. Treatment with niclosamide in vivo clearly reduces the levels of S100A4, α-SMA, and PDGFRβ in the spinal cord, as well as inflammation in central and peripheral nervous tissues, together with axonal impairment. These data are consistent with the in vitro results, demonstrating the anti-inflammatory and anti-fibrotic properties of niclosamide toward activated CNS glial cells, such as microglia and astrocytes [36, 59], and toward ALS-activated fibroblasts. Overall, these results show that niclosamide can control the excessive gliogenic/fibrotic environment and enhance neural repair in vivo in the hFUS model of ALS. Interestingly, skeletal muscles of hFUS mice display a strong increase in S100A4 expression, accompanied by augmented levels of α-SMA, PDGFRβ, and STAT3, all proteins that have been widely demonstrated to be involved in muscle fibrosis and atrophy in both mutant SOD1 mouse models and in ALS patients [17, 32]. We have shown here that niclosamide displays positive effects also on muscle atrophy by promoting muscle regeneration and inhibiting muscle fibrosis, indicating that the targeting of multiple pathways in addition to S100A4 such as mTOR, STAT3, and NF-kB, can affect disease also in muscle tissue.

Our findings deserve further research to validate this new mechanism of action of niclosamide in preclinical experiments, performing dose-response treatments and testing the drug in additional ALS models, besides FUS mice, recapitulating key pathologies and biological processes seen in sporadic ALS [3], as, for instance, the typical hallmark of TDP-43 mislocalization [62].

In conclusion, our findings show that S100A4 plays an important role in ALS-related mechanisms, and suggest that the use of a pleiotropic compound such as niclosamide, capable of affecting inflammatory, autophagic, and profibrotic mechanisms in several tissues of an ALS model, can meet the requirements of a possible treatment for ALS, that necessarily must be multifunctional and multitarget.

## Supplementary Information

**Additional file 1.**

## Data Availability

Data sharing is not applicable to this article as no datasets were generated or analyzed during the current study.

## Abbreviations

ALS: Amyotrophic lateral sclerosis
ECM: Extracellular matrix
fALS: Familial ALS
GFAP: Glial fibrillary acidic protein
hFUS: Human FUS
mTOR: Mammalian target of rapamycin
NDS: Normal donkey serum
NF-κB: Nuclear factor-κB
Non-Tg: Non-transgenic
PDGFRβ: Platelet-derived growth factor receptor β
PFA: Paraformaldehyde
α-SMA: α-Smooth muscle actin
SQSTM1/p62: Sequestosome 1
sALS: Sporadic ALS
STAT3: Signal transducer and activator of transcription 3
TGF-β: Transforming growth factor β
uvALS: Fibroblasts from ALS patients with no pathogenic variants in known ALS-associated genes
