# Supplementary material for: Targeting S100A4 with niclosamide attenuates inflammatory and profibrotic pathways in models of amyotrophic lateral sclerosis
Source: J Neuroinflammation. 2021 Jun 12;18:132. doi: 10.1186/s12974-021-02184-1 (PMC8196441; doi:10.1186/s12974-021-02184-1)
Supplement: Supplementary file 1 — Additional file 1. [file 12974_2021_2184_MOESM1_ESM.docx]

**Targeting S100A4 with niclosamide attenuates inflammatory and profibrotic pathways in models of amyotrophic lateral sclerosis**

**Authors and affiliations**

Martina Milani^1^, Eleonora Mammarella^1^, Simona Rossi^2^, Chiara Miele^1^, Serena Lattante^3,4^, Mario Sabatelli^5,6,7,^ Mauro Cozzolino^2^, Nadia D’Ambrosi^1*^, Savina Apolloni^1*^

^1^Department of Biology, University of Rome "Tor Vergata", 00133 Rome, Italy; ^2^Institute of Translational Pharmacology, CNR, 00133 Rome, Italy; ^3^Unità Operativa Complessa di Genetica Medica, Fondazione Policlinico Universitario A. Gemelli IRCCS, 00168 Rome, Italy; ^4^Sezione di Medicina Genomica, Università Cattolica del Sacro Cuore, 00168 Rome, Italy; ^5^Unità Operativa Complessa di Neurologia, Fondazione Policlinico Universitario A. Gemelli IRCCS, 00168 Rome, Italy; ^6^Centro Clinico NEMO, Fondazione Policlinico Universitario A. Gemelli IRCCS, 00168 Rome, Italy; ^7^Sezione di Neurologia, Università Cattolica del Sacro Cuore, 00168 Rome, Italy.

**Running title**

Targeting S100A4 with niclosamide ameliorates ALS

***Corresponding authors**

Nadia D’Ambrosi, PhD and Savina Apolloni, PhD

Department of Biology

University of Rome "Tor Vergata"

Via della Ricerca Scientifica, 1

00133 Rome, Italy

Tel: +39 0672594244

Email: [nadia.dambrosi@uniroma2.it](mailto:nadia.dambrosi@uniroma2.it)

[savina.apolloni@uniroma2.it](mailto:savina.apolloni@uniroma2.it)

**Supplementary Material**

**Supplementary Figure S1**

**S100A4**

**GAPDH**

**-11 kDa**

**-36 kDa**

**Ctrl#1**

**Ctrl#2**

**Ctrl#3**

***FUS p.R521C***


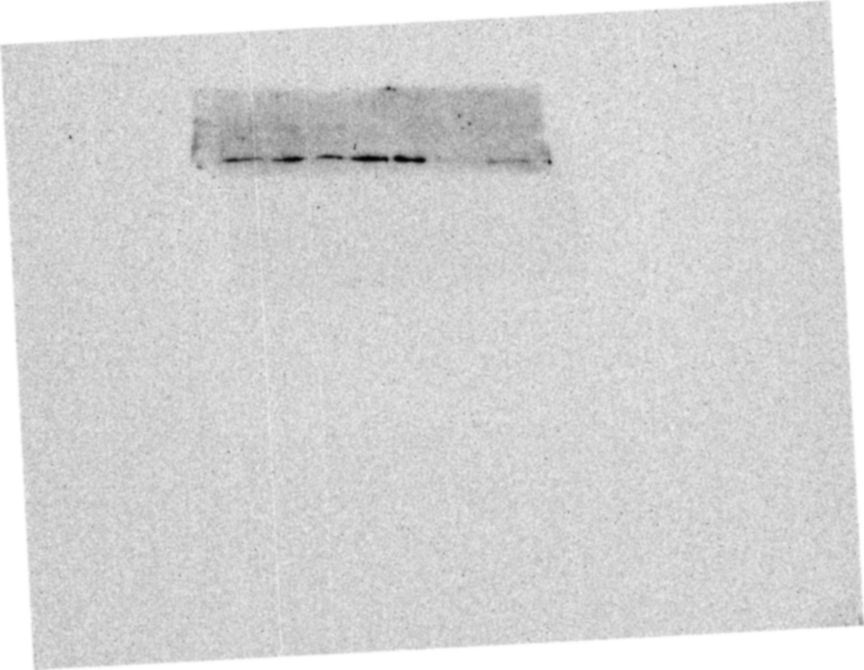

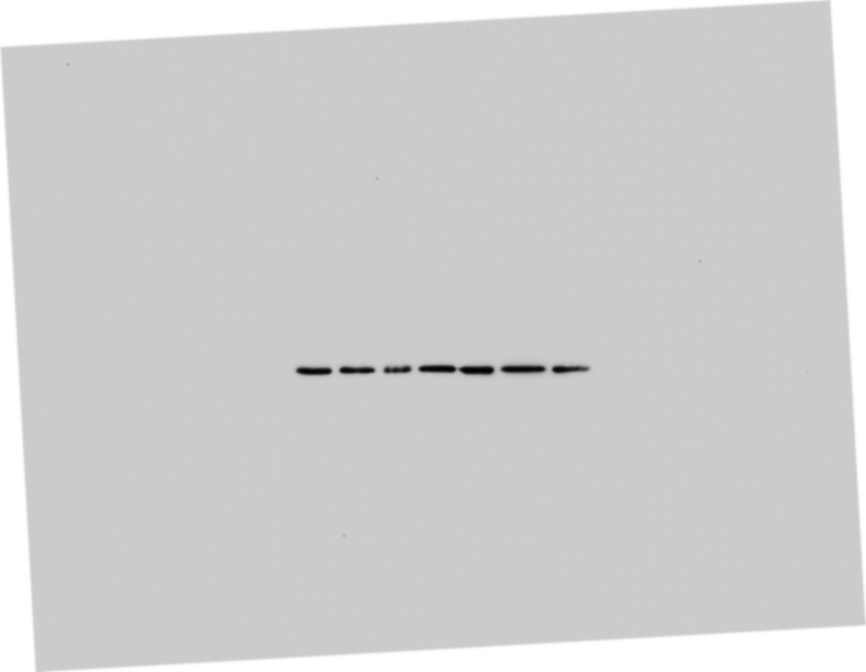


**a**


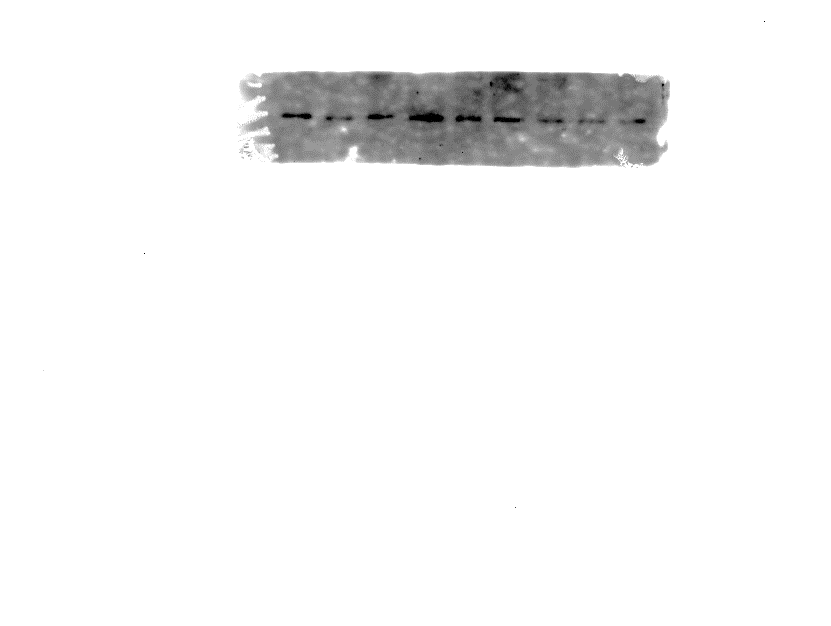


**Ctrl#1**

***TARDBP p.A382T***

**Ctrl#2**

**Ctrl#3**

***TARDBP p.Q303H***

**S100A4**

**GAPDH**

**-11 kDa**

**-36 kDa**


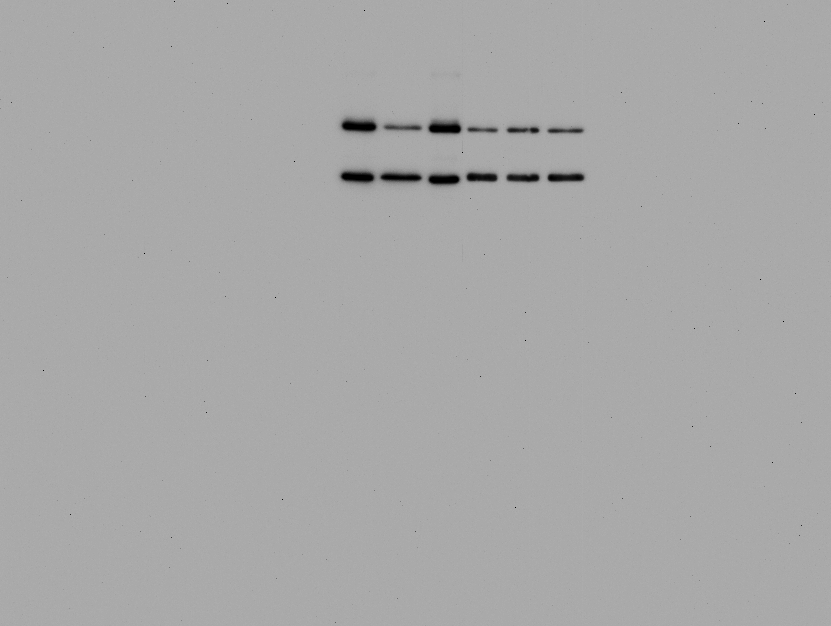


**b**

**S100A4 (AU)**

**S100A4 (AU)**

**Ctrl**

**FUSR521C**

**Ctrl**

**TARDBP**

**Figure S1. *FUS* and *TARDBP* pathogenic variants induce an upregulation of S100A4.** Protein lysates of fibroblasts from Ctrl (n=5 individuals), and from one patient carrying the *FUS* p.R521C pathogenic variant (**a**) or from patients carrying *TARDBP* p. Q303H and p.A382T pathogenic variants (n=2 individuals) (**b**) were analysed by western blotting using anti-S100A4. GAPDH was used to normalize samples. The expression levels were calculated by densitometric analyses. Data represent mean ± SEM for Ctrl and TARDBP fibroblasts.

**Supplementary Figure S2**


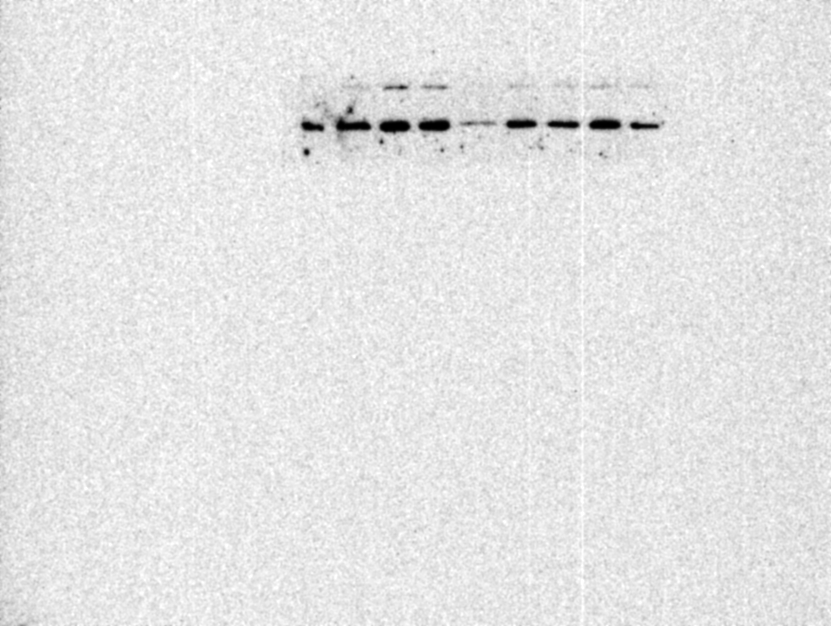


**S100A4**

**-11 kDa**

**Non-Tg**

**hFUS**


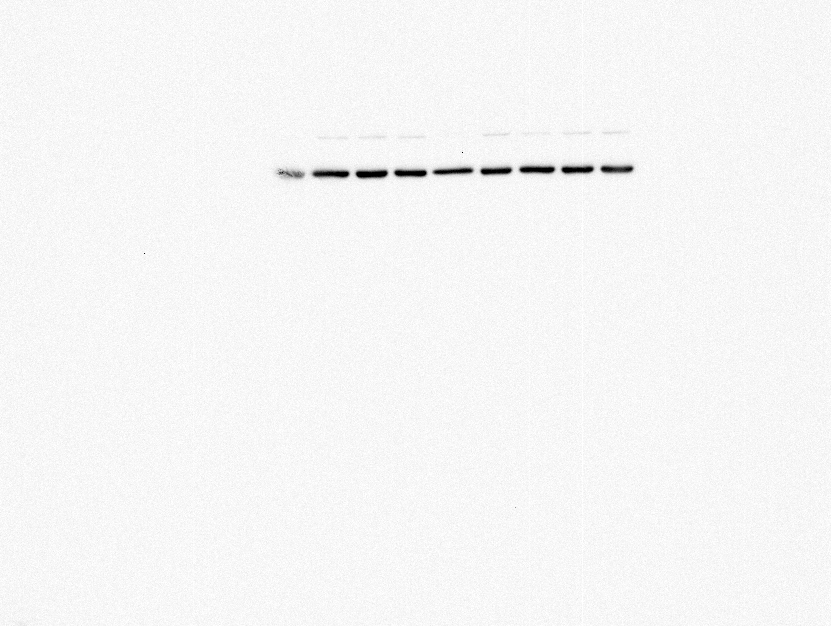


**GAPDH**

**-36 kDa**

**

**S100A4 (AU)**

**Merge**

**S100A4**

**GFAP**

**a**

**b**

**hFUS WM**

**Non-Tg**


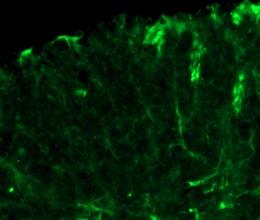

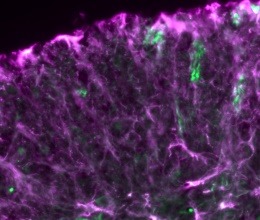

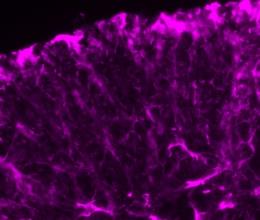

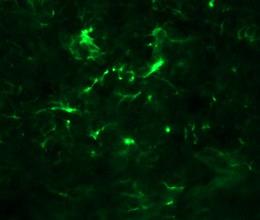

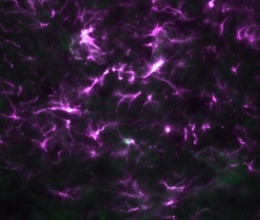

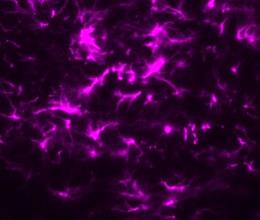

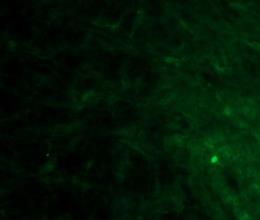

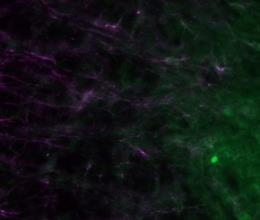

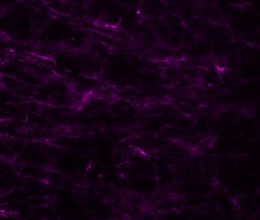


**hFUS GM**

**hFUS**

**Non-Tg**

**Figure S2. S100A4 is increased in the spinal cord of hFUS mice**. (**a**) Protein from lumbar spinal cord lysates of non-transgenic (Non-Tg) and hFUS mice at end stage (n=5/group) were assayed by Western blot with anti-S100A4. GAPDH levels served as loading control. Relative densitometric values are reported on the right. Data represent mean ± SEM. two-tailed Mann-Whitney test. ⃰⃰⃰ ⃰⃰⃰ ⃰⃰⃰p<0.01 *vs* Non-Tg mice. (**b**) Representative fluorescence images of S100A4 (green) and GFAP (purple) in grey and white matter of the lumbar spinal cord from non-transgenic (Non-Tg) (~ 40 days) and end stage hFUS mice. WM = white matter. GM = grey matter. Scale bars: 50 µm.
